# Utilizing the “Plan, Do, Study, Act” Framework to Explore the Process of Curricular Assessment and Redesign in a Physical Therapy Education Program in Suriname

**DOI:** 10.3389/fpubh.2017.00069

**Published:** 2017-04-10

**Authors:** Jennifer Gail Audette, Se-Sergio Baldew, Tony C. M. S. Chang, Jessica de Vries, Nancy Ho A Tham, Johanna Janssen, Andre Vyt

**Affiliations:** ^1^University of Rhode Island, Physical Therapy, Kingston, RI, USA; ^2^Anton de Kom Universiteit van Suriname, Physical Therapy, Paramaribo, Suriname; ^3^Elon University, Elon, NC, USA; ^4^Artevelde University College, University of Ghent, Ghent, Belgium

**Keywords:** developing nation, physical therapy, education, curricular design, teaching

## Abstract

**Purpose:**

To describe how a multinational team worked together to transition a physical therapy (PT) educational program in Paramaribo, Suriname, from a Bachelor level to a Master of Science in Physical Therapy (MSPT) level. The team was made up of PT faculty from Anton De Kom Universiteit van Suriname (AdeKUS), the Flemish Interuniversity Council University Development Cooperation (VLIR-UOS) leadership, and Health Volunteers Overseas volunteers. In this case study, the process for curricular assessment, redesign, and upgrade is described retrospectively using a Plan, Do, Study, Act (PDSA) framework.

**Method:**

PT educational programs in developing countries are eager for upgrade to meet international expectations and to better meet community health-care needs. An ongoing process which included baseline assessment of all aspects of the existing bachelor’s program in PT, development of a plan for a MSPT, implementation of the master’s program, and evaluation following implementation is described.

**Conclusion:**

Curricular assessment and upgrade in resource-limited countries requires the implementation of process-oriented methods. The PDSA process is a useful tool to explore curricular development. The international collaboration described in this paper provides an example of the diligence, consistency, and dedication required to see a project through and achieve success while providing adequate support to the host site. This project might provide valuable insights for those involved in curricular redesign in similar settings.

## Background

In developing nations around the world, the rehabilitation needs of persons with acute and long-term disability often go unmet. This problem is most often due to a combination of factors; (1) lack of understanding of rehabilitation professions, (2) inadequate numbers of rehabilitation providers, (3) inadequate infrastructure that limits access to care, (4) lack of education about the importance of rehabilitation, and (5) social constructs that inhibit people from seeking services. Physical therapists (PTs) are key providers who can directly meet the unmet clinical needs and also influence the status of the profession and the community’s understanding of physical rehabilitation. The purpose of this paper is to describe the process of curricular assessment, redesign, and upgrade of the Physical Therapy (PT) Program at Anton De Kom Universiteit van Suriname (AdeKUS) in Paramaribo, Suriname, through the retrospective lens of the Plan, Do, Study, Act (PDSA) framework.

Physical therapy curricular planning and evaluation play important roles in the advancement of the profession. This planning and assessment process is part of any PT educator’s purview. However, educational programs in developing countries, eager to enhance their capacity for being able to meet community needs and to enhance the status of the PT profession, struggle in this area because of limited expertise, resources, infrastructure, and competing priorities. Various initiatives are underway around the world to empower PTs and educational programs that aspire to advancing the profession. This paper is an example of one such effort.

## Rationale

In the recently released policy statement on PT education around the world, the World Confederation of Physical Therapy (WCPT) encourages and supports not only the implementation of appropriate entry-level education but also the development of “… processes that independently validate and assess the standards of entry-level education provision …” (1). The WCPT states that these guidelines “provide a framework for internal quality assurance processes” but does not specifically address how to develop those processes (1). This WCPT document, along with the *WCPT Guidelines for Physical Therapist Professional Entry-Level Education* (2) and the *WCPT Guidelines for Qualifications of Faculty for Physical Therapist Professional Entry-Level Educational Programmes* (3), bring to the forefront the need for upgrading and equalizing PT education practices around the world.

Using a case report format, this paper utilizes Deming’s PDSA process (4) to describe the activities and has undertaken to advance—from a Bachelor of Science level to a Master of Science in Physical Therapy (MSPT) level—of the PT education program at Anton de Kom Universiteit van Suriname (AdeKUS), an academic program providing entry-level PT education. The PDSA process reflects the reiterative and ongoing process by which the program was evaluated, redesigned, assessed, and reassessed, to achieve targeted outcomes. Outcomes of the process—rather than student outcomes—are described.

### Suriname

The Republic of Suriname is the smallest country (63,037 square miles, population 543,000) in South America (5), and 45% of the population lives in Paramaribo, the capital city.

Suriname is one of the most ethnically and culturally diverse countries in the world with a population made up of several distinct ethnic and religious groups. Dutch is the official language of Suriname, but several other languages are spoken. Education is compulsory for ages 6–12 years and the literacy rate is nearly 95% (6).

### Anton De Kom Universiteit van Suriname

Anton de Kom Universiteit van Suriname (AdeKUS), established in 1968, is the only university in Suriname and is located in the capital, Paramaribo. The medical sciences school was established in 1969 and the PT program in 1996. In 2006, AdeKUS sought funding and support from the Flemish Interuniversity Council University Development Cooperation (VLIR-UOS) (7) to enhance its medical and PT programs. Ultimately, in 2007, the PT Program was awarded funding for a 10-year project. The main goals of the grant were curriculum development and design, the upgrading of academic staff, and the upgrade of research and training facilities. In addition to those goals, it was expected that research and service areas and programs would also be enhanced in ways that would benefit the community and would promote PT in general and the MSPT program specifically.

AdeKUS, in consultation with the VLIR-UOS, determined that to best serve the changing health needs of the Surinamese population and elevate the presence and status of PTs and PT education—in the country and the Caribbean Region—the existing Bachelor program needed to be evaluated, updated, and transitioned to the MSPT level. This transition aligns with international recommendations put forth in the Bologna Process (8) and those adopted by the WCPT in the European Qualifications Framework (9). Further, AdeKUS was interested in pursuing accreditation. They realized that these efforts were necessary to increase the number of PTs, enhance the status of the profession, and meet the needs of underserved people in Paramaribo and those who live in remote areas of the country.

### Multinational Collaboration

In 2008, a multinational, collaborative effort began with involvement and contributions from the AdeKUS administrators and PT faculty, representatives of the VLIR-UOS, and Health Volunteers Overseas (HVO) volunteers from the United States (US). The work group, referred to as Team 6, met regularly in Suriname from 2008 through 2013. This team was comprised of the following people who made the following contributions:

(1) AdeKUS PT program faculty members provided information about the existing program and vision for future MSPT level program; described needs of the program, faculty, students, and community; provided ongoing feedback and program needs/outcome data; and were instrumental in implementing all aspects of the plan.
(2) VLIR-UOS project leaders led and scheduled meetings; set strategic plan goals and objectives; and met and coordinated the exchange of information with AdeKUS leadership.
(3) HVO volunteers provided knowledge and direction related to course planning, design, content, and teaching strategies to align with international guidelines; considered and communicated PT-specific accreditation expectations; instructed courses; provided clinical education training; and delivered continuing education to community therapists.

### Performance Improvement Models

**Figure 1:**
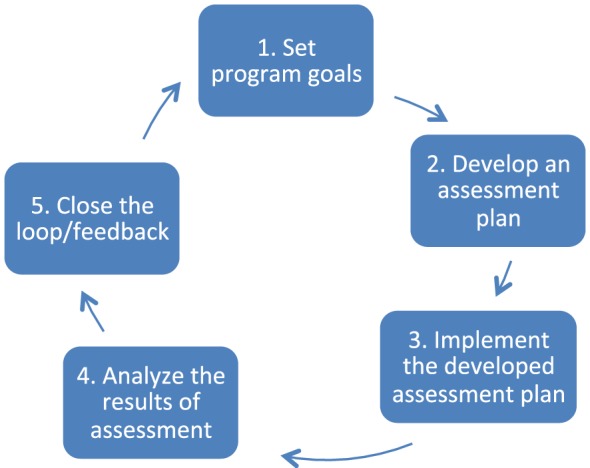
**American Physical Therapy Association outcomes assessment process for physical therapy education (14)**.

In the US, performance improvement models are routinely used in health-care organizations and PT professional education to measure, assess, and improve performance. This is likely due to the strong influence of standard setting bodies such as The Joint Commission on Hospital Accreditation (10) and the Commission on Accreditation of Physical Therapist Education (CAPTE) (11). These performance improvement models are intended to be evaluative, continuous, and reiterative. Various models have been suggested and utilized to assess PT education. One such model is the American Physical Therapy Association (APTA) model for outcomes assessment in PT education (12), based on the work of Donabedian (13). The purpose of this model is to improve the educational program through assessments of student learning and development, the curriculum, and alignment with the program’s institutional mission. Key to this assessment process is its ongoing nature, which combines the analysis of results with continuous evaluation/re-evaluation and change, which provides direction for future assessment. With descriptions of expected outcomes, indicators of progress, targets, and thresholds established, the process uses a variety of stakeholder input and “authentic and available artifacts” to determine progress and direction for change. The APTA model consists of the following components (Figure 1): goal setting; assessment planning; developing and implementing the plan; analyzing the assessment results; and closing the loop with feedback and follow-up.

In the WCPT Guidelines for Physical Therapist professional entry-level education, this important international organization encourages and supports not only the implementation of appropriate entry-level education but also the development of processes to explore and validate what is done (2). The WCPT states that these guidelines “provide a framework for internal quality assurance processes” (Section 1.4: Application) but does not specifically address how to develop those processes.

Versions of the Deming/Shewhart Cycle (Figure 1), such as the Plan-Do-Study-Act (PDSA) model, have been a part of business and industry’s quality improvement tools for decades and are based primarily on the science of improvement (14). Although the application of this model is less common for academic health-care programs, it mirrors the key aspects of the APTA model, meets the expectations put forth by WCPT, and was the model chosen to explore this particular project.

**Figure 2:**
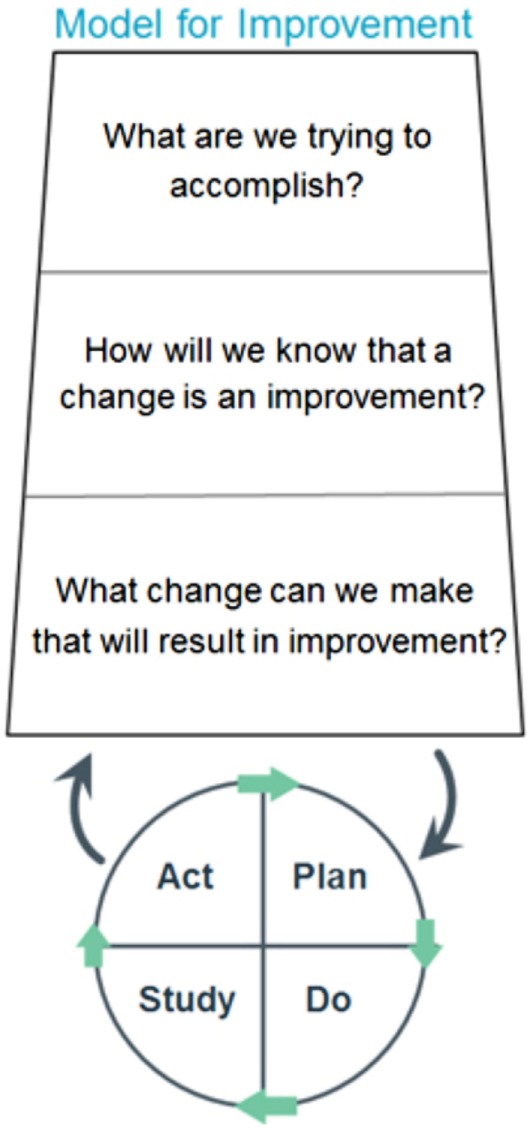
**PDSA model for improvement (13)**.

Implementing the PDSA model (see Figure 2) is useful because, when used properly, it results in an iterative process to explore the questions of: “What are we trying to accomplish?”; “What can be done that will result in improvement?”; and “How will we know there was an improvement?” Further, the model is based on taking action toward goals that are explored and assessed through the use of quantitative measures whenever possible. The involvement of those who are part of the system being improved is critical, as is the involvement of others possessing experience and expertise in the system/process being examined. In particular, the model has been used effectively in educational settings for planning, trying something, observing the results, and acting on what is learned. In essence, this is the scientific method adapted for action-oriented processes.

The cycle begins with the Plan step. This involves identifying a goal or purpose, formulating a theory, defining success metrics, and putting a plan into action. These activities are followed by the Do step in which the components of the plan are implemented. Next comes the Study step, where outcomes are monitored to test the validity of the plan for signs of progress and success or problems and areas for improvement. The Act step closes the cycle, integrating the information generated by the entire process. This can be also be used to reiteratively adjust the goals and the plan. These four steps can be repeated over and over as part of a cycle of continual improvement.

Using a case report format, this paper describes how the PDSA process was utilized during the evaluation of the AdeKUS PT program. Through a reiterative and ongoing process, the program was redesigned, assessed, and reassessed, with designated follow-up activities to achieve targeted program outcomes.

## Summary of Activities

In March of 2008, Team 6 met for the first time in Suriname. Over the next 5 years, Team 6 met regularly to address the goals of the VLIR-UOS grant to enhance the PT program by developing a MSPT curriculum, upgrading the academic staff, and upgrading the research and training facilities.

During the first meeting, the team was tasked with assessing and evaluating the structure, leadership, curriculum, and resources of the PT program and developing a general plan for upgrading the program to the MSPT level. Table 1 provides an overview of the major areas of concern and specific needs identified during the initial Team 6 meeting. These areas of need drove the PDSA process and the activities that occurred during 1- to 2-week long, on-site meetings that took place in March 2008, October 2008, March 2009, September 2011, October 2012, and October 2013.

**Table 1:** **Major areas of concern**.

Area of concern	Specific needs
Program structure	Clarification of Vision and Mission. Improve clarity of program handbooks, policy and procedure manuals, syllabi and materials, and departmental reports. Assess and clarify faculty roles and administrative structure. Regular faculty meetings. Improved program coherence and coordination. Upgrade current faculty credentials to Master of Science (MS) and Ph.D. level. Increased number of qualified faculty. Define admission prerequisites and requirements.
Program management	Define clear and specific requirements for current, new, and prospective students (prerequisites, starting level of curriculum, admission criteria). Identify benchmarks for program goals and structure. Complete community/country needs assessment.
Quality and outcome measurement	Define the current student body (#, demographics, length of time in program, practice interests, admission issues, prerequisite work). Create program for measurement of student outcomes. Measure quantity, quality, and oversight of clinical education placements. Set quality standards for teaching and learning. Aggregate data and use for planning.
Curriculum content	Align course content with contact hours. Achieve appropriate balance of basic science content and application/physical therapy (PT) focused content. Connect instructional models with course content. Explore student assessment models and develop a cohesive plan for matching assessment types to course content and learning objectives. Integrate evidence-based practice concepts. Reduce student load (particularly in the basic sciences).
Curriculum organization	Implement PT basics earlier in the curriculum. Decrease reliance on medical school faculty and curriculum.

In January 2009, information exchange experiences occurred and selected AdeKUS PT faculty visited the academic, clinical, and research laboratories of the Belgian partner institutes for three main purposes: (1) to prepare for establishing a multidisciplinary training and research center at AdeKUS; (2) to discuss quality and outcome measurement strategies for curricular, professional, and course-related issues; and (3) to explore opportunities for additional Belgian collaboration.

Team 6 did not meet in Suriname during 2010; however, during that year, continued communication, support, and volunteer teaching took place. Various members of Team 6 visited AdeKUS individually to carry out a variety of supportive activities and consultation such as: teaching, document revision assistance, continuing education for faculty and community clinicians, and clinical instructor training.

During each Team 6 meeting a micro-level iterative process of planning, doing, studying, and acting on findings took place. Although providing specific details from each of those meetings is beyond the scope of this paper, Table 2 provides a macro-level view of how the PDSA process worked over the 6 years. The table includes thematic categories of issues and activities that were identified, addressed, and accomplished. Information is included in relative chronological order. Several of the activities occurred a number of times because new need arose or the issue required exploration with a different focus or more depth. Certain items remain ongoing because they are vital to keeping the process fluid and dynamic. Those are discussed in the category specific narratives below. The items included in Table 2 are included because they are likely pertinent to other programs planning or undertaking curricular upgrades. Some explanation for each of the major themes is provided below.

**Table 2:** **Summative Overview of Plan, Do, Study, Act Issues, Activities, and Accomplishments**.

**Plan**	Establish Team 6 Assess and evaluate existing Bachelor of Science in Physical Therapy (PT) program Develop Master of Science in Physical Therapy (MSPT) program Develop and plan for all aspects of transition Establish a Training and Research Center and laboratory Provide regular updates to AdeKUS leadership								

**Issues and activities**		**Accomplished**							
		**March 2008**	**October 2008**	**January 2009**	**March 2009**	**2010**	**September 2011**	**October 2012**	**October 2013**

**Do**	**Program structure and leadership**								
	Gather information about existing program structure and leadership	**x**	**x**						
	**Curriculum**								
	Gather information about existing BS in PT curriculum	**x**	**x**						
	Develop a framework for the new MS in PT program		**x**		**x**				
	Admit MSPT students					**x**	**x**	**x**	**x**
	**Faculty and Professional Development**								
	Assess faculty development needs		**x**						
	Provide professional development opportunities for faculty, students, and local clinicians			**x**	**x**	**x**	**x**	**x**	**x**
	**Resources**								
	Evaluate existing and necessary resources (human, material, and space)		**x**	**x**	**x**				
	**Outcome assessment**								
	Establish an outcome measurement process				**x**				
	**Establish Team 6**								
	Determine membership and roles	**x**							**x**

**Study**	**Program structure and leadership**								
	Program guiding documents	**x**	**x**						
	Program management		**x**						
	Admission process and criteria for entry into MSPT part of the program				**x**				
	Need for a PT education board							**x**	
	Advising processes						**x**		
	Leadership clarity, roles, and responsibilities		**x**		**x**		**x**		
	**Curriculum**								
	MSPT program development				**x**				
	Curriculum content and organization			**x**	**x**				
	First years of MSPT					**x**			
	Plan for subsequent years of MSPT						**x**	**x**	**x**
	**Faculty and professional development**								
	Faculty development needs				**x**				
	Faculty job descriptions and curricular responsibilities		**x**						
	Potential interest in Master of Science for Suriname physical therapists (PTs)				**x**				
	Potential interest in Transitional Masters for previously trained PTs							**x**	
	**Resources**								
	Educational resource needs			**x**					
	Space available for training and research			**x**					
	**Outcome assessment**								
	Identify existing processes		**x**						
	Create new process				**x**				
	Program strengths and weaknesses on an initial and ongoing basis	**x**					**x**	**x**	**x**
	**Program accreditation**								
	Potential for program accreditation								**x**

**Act**	**Program structure and leadership**								
	Strengthen program structure			**x**					
	Enhance program management			**x**			**x**		**x**
	Clarify and strengthen leadership						**x**	**x**	**x**
	Established an educational board and defined responsibilities								**x**
	**Curriculum**								
	Assess existing and needed curricular resources	**x**	**x**	**x**			**x**		
	Solidify MSPT curriculum content and organization			**x**		**x**			
	Address student prerequisite issues						**x**		
	Rectify imbalances in the curriculum			**x**				**x**	
	**Faculty and professional development**								
	Identify and interview current faculty and community therapists interested in pursuing advanced degrees abroad				**x**		**x**		
	Solicit input from community clinicians about desire for t-MPT, continuing education courses, specialty certification				**x**			**x**	
	Decide to continue to use MD faculty until PT department faculty can meet all program teaching and expertise needs				**x**		**x**		
	Provide faculty training about pedagogy and student assessment				**x**		**x**		
	Make MSPT available to community therapists						**x**		
	Discuss involvement in international committees and organizations								**x**
	**Resources**								
	Identify specific resource needs	**x**							
	Identify space for a research center and laboratory				**x**				
	Identify funding for equipment and operational costs					**x**			
	Identify need and create a plan for utilizing international volunteer teachers		**x**			**x**	**x**		
	**Outcome assessment**								
	Institute outcome assessment and management program					**x**			
	Assesses initial years of MSPT program						**x**	**x**	**x**
	Utilize data to plan for future						**x**	**x**	**x**
	**Accreditation**								
	Address desire to seek accreditation for PT program								**x**
	**Team 6**								
	Revise and adjust Team 6 member roles and involvement								**x**
	**Other**								
	Meet with AdeKUS leadership to report findings, suggestions, plans	**x**	**x**	**x**	**x**	**x**	**x**	**x**	**x**
	Celebrate, share information, and enhance recognition of the program						**x**	**x**	**x**

### Program Structure and Leadership

The organizational structure of the program was examined in relation to PT education programs in Europe and the US. Guiding documents, program handbooks, policy and procedure manuals, committees, and meeting schedules were assessed for existence and quality. This resulted in the development, revision, and organization of the Mission and Vision statements, program handbooks, and policy and procedure documents. It also prompted the clarification of leadership roles and responsibilities, which was important for the day-to-day running of the program, but also served to empower those who had leadership expectations. This included the identification of a Program Director and gave him/her power and time to carry out leadership and administrative tasks. Importantly, this person had a direct supervision line to the Dean. A calendar of regular program, faculty, chair, and dean meetings was established.

A community/country needs assessment was carried out to determine PT service needs and the needs of community clinicians. This information led to the discussion and enhancement of program goals and expected outcomes. This included the identification of benchmarks for outcomes and performance, the development and revision of information about student admission prerequisites and requirements, and other informative documents for new and prospective students. It also led to the development of a transitional masters program for previously trained community therapists.

Initially the program was heavily reliant on medical school faculty who taught major aspects of the PT curriculum. This was problematic in two particularly important ways. First, the PT program had no autonomy or control over what was being taught and lacked the ability to hold those instructors accountable for what they did. Second, the students were learning from a medical perspective and not a PT perspective. Even within the PT program there was a need for clarification of roles and responsibilities. Some faculty had very heavy teaching loads while some had very light teaching loads. In some areas, expertise was lacking and the program had a reliance on international volunteers to come and teach courses. It was identified that many faculties would benefit from education and training to enhance pedagogical practice, teaching methods, and student assessment techniques.

These issues led to the development of a curriculum that was less reliant on the medical school faculty, a plan to progressively shift to more Master of Science (MS) and Ph.D. trained faculty, involvement of community PTs as lab and teaching assistants, a plan and schedule for regular faculty and committee meetings, continuing education to enhance teaching methods and strategies, and evaluation of teaching loads and expectations. Job descriptions were developed for all faculty and staff.

Later in the process, a Faculty Board was put in place. The Board would have responsibility and clout in addressing; curricular issues, outcome measurement results, student assessment, coordination, faculty performance, program evaluation, faculty assignments, research expectations, admission criteria, faculty recruitment, and space and resources. The Faculty Board was made up of PT program faculty and students, faculty associated with, but not a part of the PT program, and community PTs. The Board was tasked with establishing a regular meeting plan before start of each academic semester, run by the Program Director, where program faculty meet to discuss curriculum, send a consistent message to students about curriculum and course work, and stimulate collaboration. Importantly, the Board would also be responsible for the future management and approval of newly developed syllabi and course changes. This process would include syllabi acquisition, assessment, and revision. Subgroups of the Board would be responsible for specific didactic issues including specific faculty issues, course content, and incorporating evidence-based practice.

The development of leaders with the appropriate skills was and remains an ongoing challenge. There have been several changes in the leadership roles and responsibilities throughout the process.

### Curriculum

Evaluation of program curriculum was carried out in relation to the Physical Therapy Normative Model, accreditation standards from CAPTE, and evaluative criteria from the Netherlands Flemish Accreditation Organization (NVAO) (15). Although the exact standards differ between the organizations, there are consistent themes and expectations that resonate across resources. Those themes were used to evaluate specific areas of the AdeKUS PT curriculum and to identify areas of strength, need, and opportunities for enhancement. Information was gathered from documents, AdeKUS faculty, recent graduates, administrators, community leaders, and current students.

At the initial meeting in 2008, the team was informed that it was taking students a very long time to complete the PT curriculum; the team thought this was the result of an inability to enlist international volunteer instructors in certain content areas. However, it also became clear that this problem was due to a couple of other things. These included (1) the use of a lottery system for admission to the program and (2) the common and chronic problem of students having to retake many courses because of failure were brought to light at this meeting. The use of a lottery system for admission to the program meant that students had not chosen PT and may or may not have knowledge of—or interest in—the profession. Low pass rates were felt to be due to the practice of student performance rarely being assessed during a course. Rather, a student’s grade is dependent on one exam given 1–3 months after a course has been completed. Neither the lottery system nor the exam system could be changed, but there was opportunity to strengthen the teaching and student assessment practices and improve student advising within courses in the PT curriculum.

Based on all of these factors, several initiatives to assist students were implemented. These included a program-orientation process, an advising system, and professional development sessions. Contact hours and student workload were explored in depth, and the findings informed the development of the new curriculum. The importance of syllabi for guiding students was discussed and informative and thorough syllabi were developed. Access to important resources such as texts and other resources, Internet access, and lab space was enhanced.

**Figure 3:**
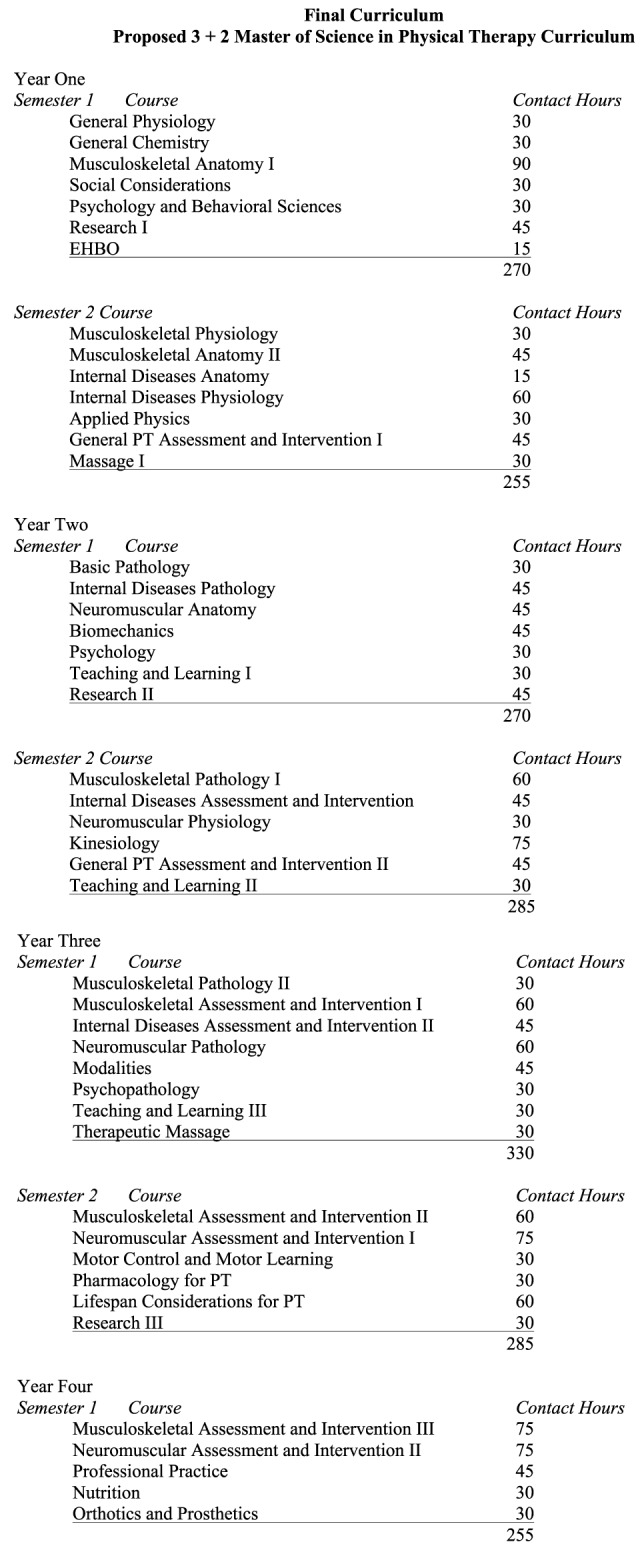

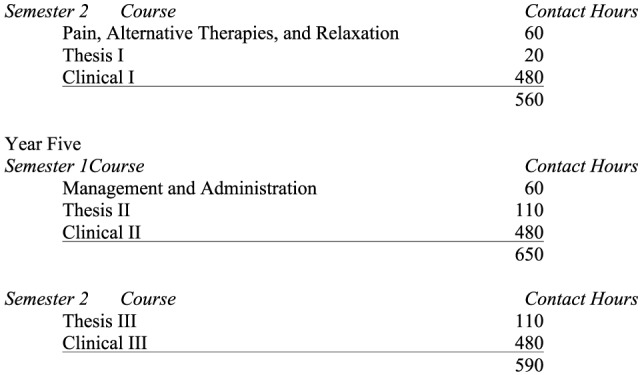
**Master of Science in Physical Therapy curriculum**.

A proposed “3 plus 2” MSPT curriculum, with the awarding of a bachelor’s degree after successful completion of the first 3 years, was developed (see Figure 3), and approval of that proposal was received from AdeKUS leadership. The curriculum was built on three main pillars; (1) musculoskeletal rehabilitation, (2) internal disorders, and (3) neuromotor rehabilitation that address domains across the lifespan, incorporate psychomotor learning topics, and integrate evidence-based practice. It was determined that in order for the new curriculum to be implemented properly and successfully several changes needed to be made. These included rectifying content imbalances in the curriculum; integrating the concept of evidence-based practice; comparing actual contact hours to US and Belgian models and proposing revised contact hours; providing faculty development opportunities related to instructional practices, student assessment practices, faculty leadership, and many other issues; involving community therapists in the curriculum and program activities; assessing and discussing overseas volunteer needs; and providing continuing education to faculty and students. A final version of the MSPT curriculum was completed following discussion and consideration of the above issues. Table 3 provides a comparison of the BS in PT and MSPT curricula.

**Table 3:** **BS and Master of Science in Physical Therapy (MSPT) curriculum comparison**.

Course	BS degree	MPT degree
	Existing contact hours	Proposed contact hours
Teaching and learning	Integrated in other courses	60
Orthotics and prosthetics	Minimally addressed	30
Basic pathology	30	30
Advanced pathology/pharmacology	180	90
Pharmacology for the physiotherapist	In pathology course	30
Professional practice	15	45
Management and administration	45	60
Nutrition	Minimally addressed	30
Pain assessment/alternative therapies	Integrated in other courses	60
General physical therapy (PT) assessment and treatment	Integrated in other courses	90
Psychology and behavior	Integrated in other courses	30
Psychopathology	30 (psychiatry)	30
Health psychology	Minimally addressed	30
Psychosocial issues	Integrated in other courses	30
Chemistry	130, Not PT specific	30
General physiology	130, Not PT specific	30
Applied physics	130, Not PT specific	30

**Musculoskeletal pillar**		

**Course**	**Existing contact hours**	**Proposed contact hours**

MS anatomy	155	135
MS physiology	In general physiology	30
Biomechanics	60	45
Kinesiology	95	75
MS assessment and treatment 1	180	60
MS assessment and treatment 2		60
MS assessment and treatment 3		75

**Internal Disorders Pillar**		

**Course**	**Existing contact hours**	**Proposed contact hours**

ID anatomy	20	15
ID physiology	In general physiology	60
Nutrition	20	30
ID pathology	In general pathology	45
ID assessment and intervention	Integrated in other courses	90

**Neuromotor Pillar**		

**Course**	**Existing contact hours**	**Proposed contact hours**

NM anatomy	45	45
NM physiology	In general physiology	30
Motor control and motor learning	In treatment and assessment	30
NM pathology	In general pathology	60
Life span course	Integrated into other courses	60
NM assessment and treatment 1	105	75
NM assessment and treatment 2		75

**Evidence-Based Practice Pillar**		

Research methodology 1	0	45
Research methodology 2	0	45
Research methodology 3	0	50
Thesis 1	0	110
Thesis 2	0	110
**Total**	About 1,370	2,190

As with any academic program, the MSPT program and its content will continue to be evaluated and revised as indicated by analysis of stakeholder feedback.

### Faculty and Professional Development

As part of the transition process, faculty development needs were evaluated and a plan to address them was developed. Initial activities to address faculty needs included the creation of job descriptions based on program needs and the assignment of curriculum Pillar Coordinators to be responsible for related courses, syllabi development, and advising course instructors to coordinate and enhance the pillar they were responsible for.

The development of the new curriculum—and the transition process itself—necessitated the upgradation of current faculty and the recruitment of additional faculty. A plan for faculty to travel abroad and earn advanced degrees was developed. Candidates interested in receiving scholarships to travel to Belgium to complete MS degrees were recruited and interviewed. Initially, candidates were chosen to study in the three curricular pillar areas mentioned previously. Once their degrees were completed, they would be hired as faculty in the new MSPT program. This opportunity was open to all PTs in Suriname. Over the years, three rounds of this process took place. The absence of the faculty who were earning degrees abroad had major detrimental impacts on the program. Various measures to alleviate the impact were put into place, including a plan for the increased use of international volunteer course instructors, long-term assistance from volunteers to assist with all aspects of the program, and the decision to not admit a class of students in 2009.

Community therapists were also included in the continuing education efforts. They too were eligible for opportunities to earn advanced degrees abroad. Locally they were included as lab assistants, attended continuing education sessions, and provided input about the program upgrade. A transitional MSPT was developed for them at their request.

Later in the process, opportunities for AdeKUS involvement in international committees and organizations and international collaborations for research and education were explored.

Current and future faculty will be encouraged to participate in professional development activities and attainment of advanced degrees in order to be able to meet the needs of the program and their own personal growth. Community clinicians will remain involved in the program in a variety of ways.

### Outcome Assessment

The team determined that a variety of items needed assessment, and benchmarks were determined. An outcome measurement process was put in place to gather baseline data and explore programmatic outcomes, faculty outcomes, teaching and learning quality, student outcomes, quality of clinical education placements, reasons for delayed graduation, and composition of the student body.

In the fall of the 2011–2012 academic year, the outcome measurement initiative was implemented. A survey of faculty and students was carried out using a set of questionnaires from the Basic Quality Scan in the PROSE toolbox (16). PROSE is a toolbox with sets of standardized questionnaires validated by quality management experts in higher education. The application of PROSE was selected as an example of best practice at the 2013 Conference of the European Organization for Quality (17). This system allows respondents to evaluate items on a rating scale and also provide comments. Quantitative and qualitative data are available, and items for improvement can be identified and prioritized. The system generates indexes by questionnaire and by item, identifies weak and strong elements, and generates an overview of priorities based on participant responses. AdeKUS faculties were asked to complete 7-topic-specific questionnaires (20 items each) which included: program design; teaching and learning methods; assessment; study load and progress; organization; staff; and quality assurance. Students were asked to complete three of the questionnaires; teaching and learning methods; assessment; and organization. Three waves of program evaluation were organized for 2011, 2012, and 2013 in which the PROSE Online Diagnostics System (16) was used. The first wave of evaluation (2011) focused on the course quality, course difficulty, and study load. Ten faculty members and nine students completed the requested surveys. The performance indexes for faculty responses ranged from 50 to 76 (index interpretation: >60 sufficient, >70 good, >80 very good, >90 excellent). The students’ ratings were substantially lower than the ratings of the faculty.

Discussion of PROSE results led to the clear identification of areas for improvement. An action plan that identified specific tasks and responsible persons was established. In an effort to limit the amount of change, it was decided that only minor changes would be made to the first year of the curriculum. In terms of the second year of the new program, the team established the following plans: continue to work to clarify faculty roles and responsibilities; attempt to improve communication between faculty members and between faculty and students; use HVO volunteers to teach courses as needed and as available; and consider evaluation of the first year and how it is relevant as the program moves through its second year.

In the two subsequent waves of evaluation (2012 and 2013), the team found many areas had improved and certain problems persisted. Strengths included overall improvement in program delivery; an increase in quantity and quality of PT-specific instructors and content; and a student perception that the workload was acceptable. The response rates were low and too limited to extract significant results; however, some important areas for improvement were evident. The most serious problem was that students continued to have trouble moving through the curriculum. This seemed to be due to lack of faculty to teach certain courses, lack of non-PT faculty connection with—and dedication to—the PT curriculum, and poor examination results. Problems also included lack of clarity about organizational structure and coordination; lack of clarity in course materials and syllabi; problematic Internet access and reliability; and faculty shortages.

Based on the results, continued syllabi revision took place: supports for students were enacted; gaps in instructor expertise and availability were addressed; Internet system upgrades were requested; and faculty were reminded to keep to the course hours and workload specified in the curriculum. The aggregated data were used on an ongoing basis for planning in all aspects of the program.

Seeking and receiving adequate and useful feedback from stakeholders will continue to be a focus, as will designing a streamlined way of collecting and analyzing the data. Efforts will take place to enhance the number of respondents. The outcome measurement process will continue to evolve so that valid information is collected from a large number of stakeholders so that follow-up efforts can be maximized.

### Resources

Educational resource needs were assessed. Necessary books, reference materials, computer access, lab space, materials, and equipment were identified, and a plan to acquire them was developed. Over the course of the project, efforts to improve Internet access were made, text books were purchased, and space and equipment for a research laboratory and a clinical laboratory/practice room was obtained. Grant funding for resources was identified.

Adequate Internet access continues to be problematic. Ongoing review of the adequacy and availability of resources will require ongoing attention as the program grows and changes and as research activities increase.

### Accreditation

Toward the end of the process, AdeKUS decided to seek local accreditation, and preparation for that became a major priority for the PT program.

## Summary

The Suriname curriculum upgrade project has been exceedingly successful, although it was not without its challenges. The most notable of which were: a lack of clarity about leadership, the need to send faculty aboard to earn advanced degrees, reliance on the medical faculty, and cultural differences with regard to urgency and timeframes. Initially, it was difficult to identify who was responsible for various aspects of the program. Further, people outside of the PT program faculty had various leadership roles. This complicated matters and made it difficult for the program to be autonomous. When faculty members traveled to Belgium to earn advanced degrees, this weakened the program structure while they were away and prevented some things from being accomplished in the most efficient ways. The reliance on the medical faculty to teach PT content was problematic. Those instructors did neither have adequate knowledge of PT content nor did they have an allegiance to the program. This created problems in terms of PT-specific curricular content, inadequate student support, and disenfranchising program leadership because they had no control over the medical faculty. Team 6 encountered cultural differences that needed to be addressed early on in the process. In particular, this included making the most beneficial use of the time the team had to work together and the expectations that tasks would be completed as assigned for subsequent meeting.

Despite these challenges, the AdeKUS PT program admitted the first class of MSPT students in November 2010. A new class of 10 to 15 students has been admitted every year since then. In the last 2 years, the program has grown in popularity so the lottery system is used to select 10 students, only after the 5 students with the top scores are admitted. Nine students have finished the MSPT.

As of now, six faculty members have completed their MS degrees in Belgium. Two faculty members plan to receive their Ph.D. degrees in 2017, while the third is planning to finish her Ph.D. in 2018. The other faculty members with MS degrees will start Ph.D. programs in the near future.

Clinical instructors are receiving continuous education to increase the quality of clinical education experiences. The program infrastructure (faculty, documents, space, resources, and expectations) has been upgraded in many ways to allow those things to occur. The most critical aspect of Team 6’s success is related to the fact that the AdeKUS PT faculty was very involved in the process and receptive to the team’s input and feedback—even when the comments were critical of past practices. The PT faculty were smart, eager, invested, and excited about the future of the program.

Evaluation of the success of the program transition is ongoing. Although Team 6 and HVO volunteers no longer meet in Suriname, members of the team continue to assess effectiveness and to make changes as needed. Once the basic needs of the program are met and running smoothly, plans include more sophisticated tracking of student demographics, qualifications, and success, which will allow for comparison with past students and other academic programs and provide rich data for ongoing programmatic evaluation. Future work will focus on coordinating and evaluating program expansion, exploring student outcomes, and consideration of the AdeKUS PT program’s role at the international level.

In summary, AdeKUS, in consultation with the VLIR-UOS and HVO volunteers, determined that to best serve the changing health needs of the Surinamese population and elevate the presence and status of PTs and PT education in the country and the Caribbean Region, the existing Bachelor of Science program needed was thoroughly evaluated against current international expectations and practice. The program was then updated accordingly and, with constant examination and assessment, transitioned to the MSPT level. This curricular upgrade and transition project aligns with international professional recommendations. The PDSA model provides a lens through which to explore the process and consider future curricular update projects in developing nations. In this case, it was used to explore how Team 6 was able to meet the initial goals of the VLIR-UOS grant. This multi-faceted, process-focused, international collaborative approach to implementing change was very successful; yet, it exemplifies the care with which such projects should be undertaken. Even with a strategic, process-oriented team of international colleagues, in a relatively controlled and stable national situation, with highly invested and motivated local faculty, some serious challenges existed and persist.

In this case, the PDSA process facilitated the identification of several important themes/issues and led to the upgrade and improvement of the AdeKUS PT program. Several of the problems identified and addressed through the process are likely relevant to most curricular upgrade initiatives. These themes and issues include thorough evaluation of existing organizational structure/practice; development of leadership skills and empowerment; consideration of the impact of student admission processes and academic readiness; deep assessment and analysis of curricular content; assessing and maximizing faculty capacity and needs for continuing education; seeking stakeholder buy-in; seeking, incorporating, and supporting the involvement of community clinicians; ensuring the availability of adequate resources; establishing outcome measurement processes; evaluating the need for outside support; and working toward a self-sustainable end. Entities interested in undertaking similar curricular development or upgrade projects—no matter the discipline—should be mindful of the many issues that might complicate their efforts, the time needed for transition, and the importance of using an improvement model that will provide structure for the project.

The international collaboration described in this paper provides an example of the diligence, consistency, and dedication required to see a project through and achieve success while providing adequate support to the host site. Although this paper describes the upgrade of a PT education program, it can be used to inform curriculum development projects in developing nations in any of the health disciplines.

## Author Contributions

JA is the primary author. S-SB assisted with writing and editing. TC contributed with writing and editing. JdV contributed with writing and editing. NT assisted with writing and editing. JJ contributed with meeting content and development of paper. AV assisted with writing, editing, and PROSE content.

## Conflict of Interest Statement

The authors declare that the research was conducted in the absence of any commercial or financial relationships that could be construed as a potential conflict of interest.
